# Supplementary material for: Contextual conditions define maximum energy-use threshold in low-carbon controlled environment agriculture for agri-food transformation
Source: Nat Commun. 2026 Feb 2;17:880. doi: 10.1038/s41467-026-68631-w (PMC12865188; doi:10.1038/s41467-026-68631-w)
Supplement: Supplementary file 1 — Supplementary Information [file 41467_2026_68631_MOESM1_ESM.pdf]

Supplementary Information for

Contextual Conditions Define Maximum Energy-use Threshold in Low-Carbon Controlled Environment Agriculture for Agri-Food Transformation.

Shiwei Ng[1,2*], Olaf Hinrichsen[1,2], S. Viswanathan[3*]

[1] TUMCREATE, 1 CREATE Way, #10-02 CREATE Tower, Singapore 138602
[2] TUM School of Natural Sciences, Department of Chemistry, Technical University of Munich, Germany
[3]Nanyang Business School, Nanyang Technological University, Singapore
*Corresponding Authors

Correspondence to: shiwei.ng@tum-create.edu.sg, asviswa@ntu.edu.sg

This PDF file includes:

Supplementary Figure 1 Emission Factors of Renewable Energy and Prospective Grids in 2050. 2
Supplementary Figure 2 Sensitivity Analysis of Maximum Criterion to Different Prospective Energy Grid Scenario. 3
Supplementary Figure 3 Maximum Criterion for Growing Lettuce and Tomato in Selected Countries 4
Supplementary Figure 4 Extracted Energy Use-Productivity Metric of Different CEA Configurations in Different Climatic Conditions to Grow Lettuce and Tomato From Literature. 5
Supplementary Figure 5 Sensitivity Analysis of Maximum Criterion to Consumption-Based, Carbon Accounting Approach (Wheat and Soy). 6
Supplementary Figure 6 Sensitivity Analysis of Maximum Criterion to Carbon Opportunity Cost Calculation Approach 7

Other Supplementary Materials for this manuscript include the following:

Supplementary Data 1: Calculated Maximum Criterion for Importing Countries.

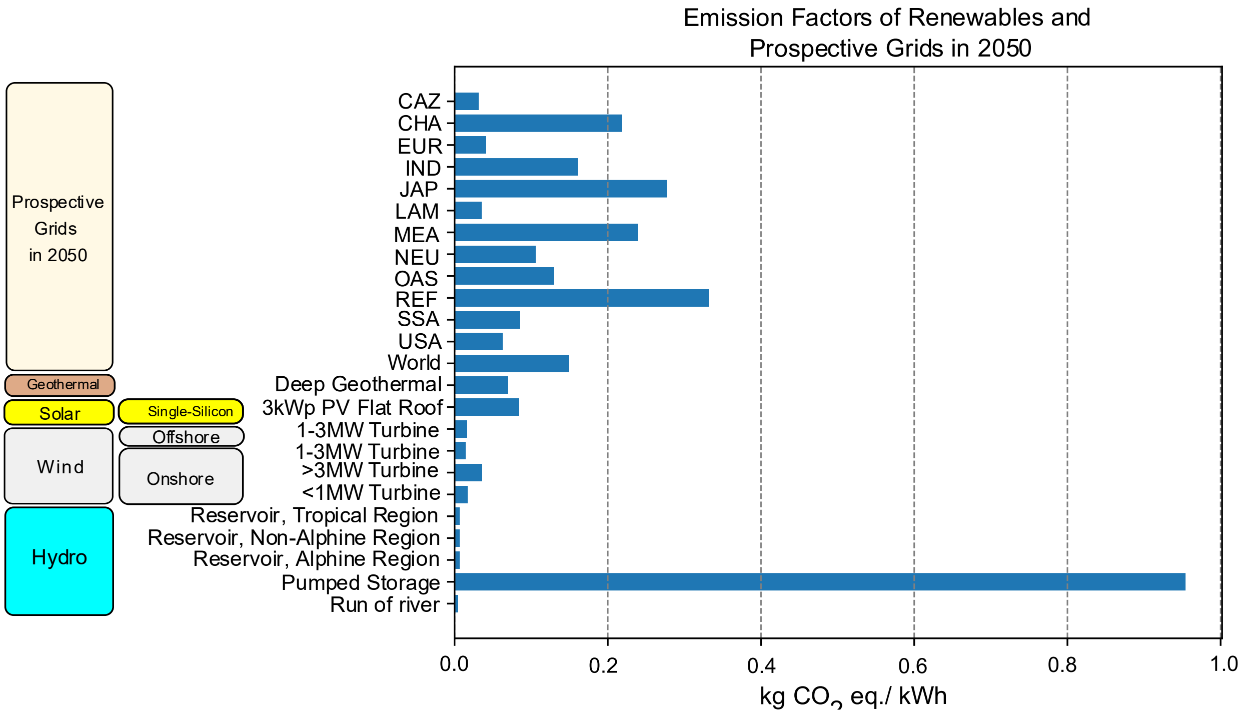

Supplementary Figure 1 Emission Factors of Renewable Energy and Prospective Grids in 2050. Geographical aggregation in REMIND consists of five individual countries (CHA-China, IND-India, JAP-Japan, USA-United States of America, and RUS-Russia) and eleven regions. Generally, emission factor of hydropower and wind powered renewable energy are the lowest, except for “Pumped Storage” in which pump electricity from non-renewable sources were assumed to be used in Ecoinvent[1]. 3 kW_{peak} single silicon photovoltaic panels have higher emissions per kWh as compared to other renewable energy.

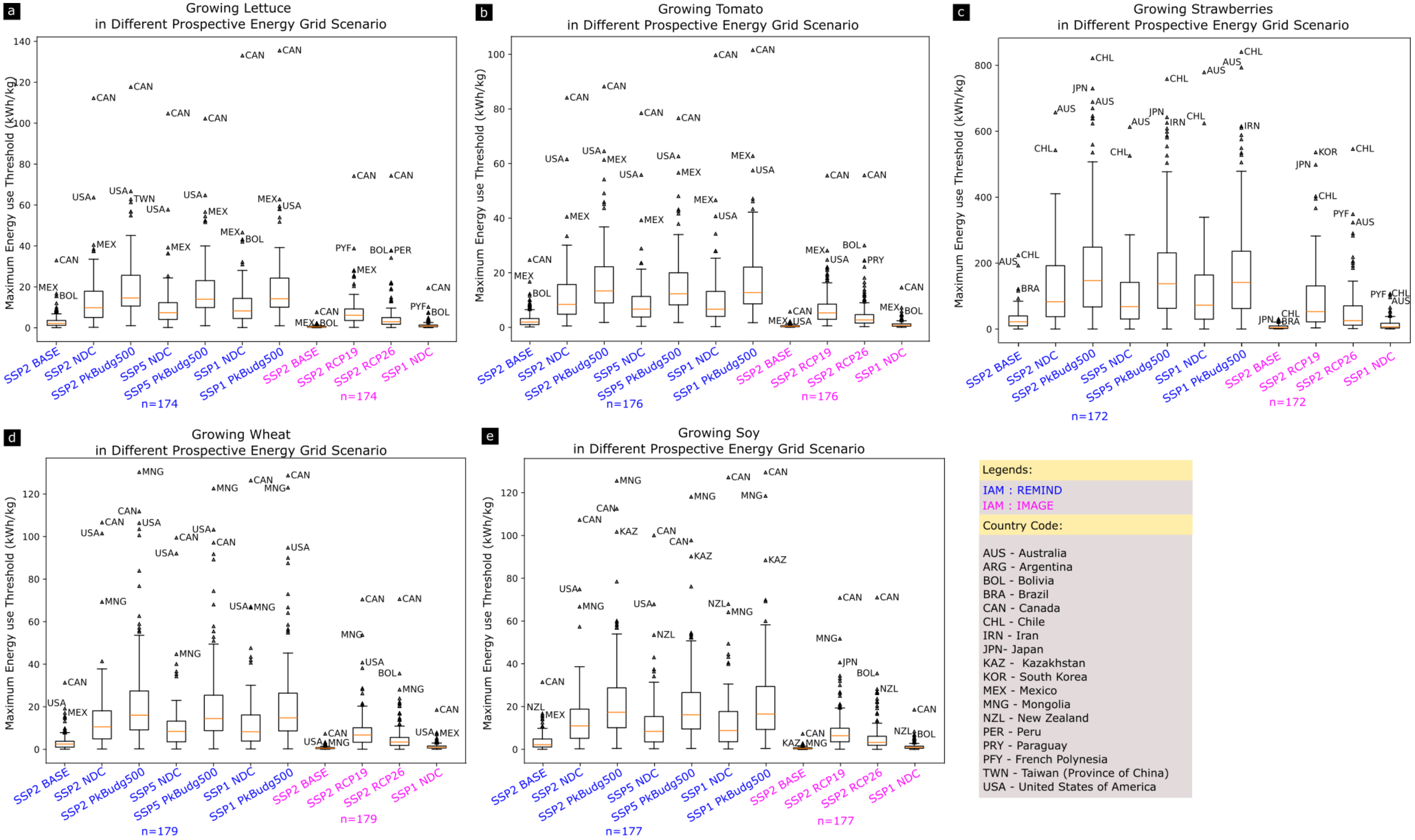

Supplementary Figure 2 Sensitivity Analysis of Maximum Energy Use Threshold to Different Prospective Energy Grid Scenario. The boxplot reflects the median (blue center bar), interquartile range (± 25% of median), whiskers (1.5 times the interquartile range), and outliers (triangle markers outside of whisker cap). Besides the "middle of the road" shared socio-economic pathway (SSP), there could be multiple transition pathways of varying resistance to mitigation and adaptation policies, as well as several representative concentration pathways (RCP) to limit global warming. Ten other analyses were conducted in addition to the SSP2 Base scenario shown in the main article. Including four scenarios using outputs from IMAGE IAM model. REMIND model illustrates more favourable energy scenarios for CEA while IMAGE model presented more stringent operational requirements. And within the outputs from REMIND models, the projected RCP influenced CEA maximum criterion more than the analysed SSP. If Nationally Determined Contributions (NDC) were to be achieved, for half of the countries importing lettuce, tomato and strawberries, CEA would likely be a viable option to achieve both food security and sustainability objectives. However, for high calorific value crops, the maximum criterion for import replacement remains an unachievable performance target for current indoor farming trials.

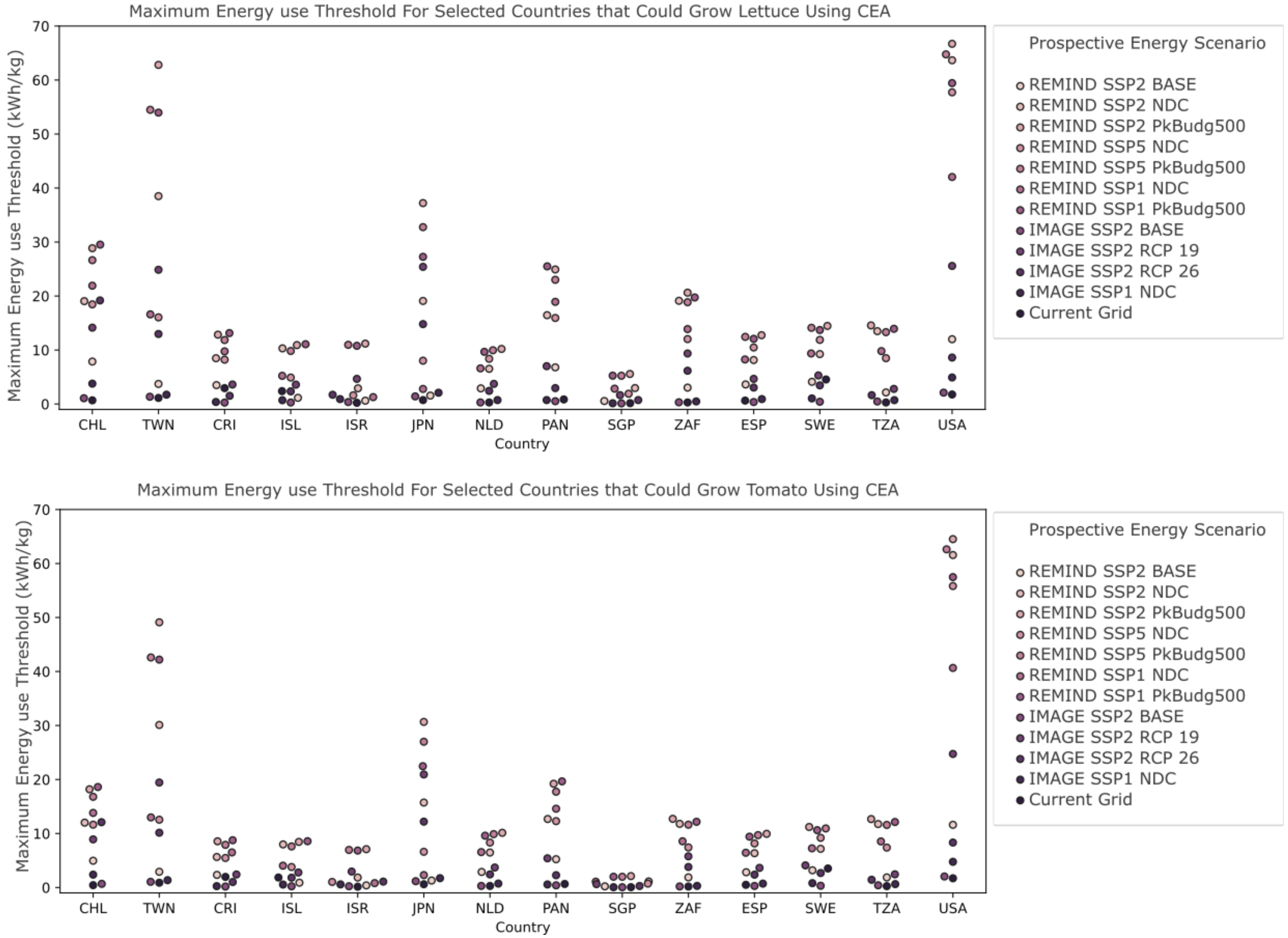

Supplementary Figure 3 Maximum Energy Use Threshold for Growing Lettuce and Tomato in Selected Countries
As seen from Supplementary Figure 2, different prospective scenarios would yield different maximum criterion for importing countries. Here, maximum criterions for selected countries with high CEA interest, as highlighted in Supplementary Figure 3, were illustrated. Comparing with the average energy use-productivity in Supplementary Figure 4, the more stringent the climate policy, the higher the maximum criterion and the more likely a CEA venture would be able to operate within this threshold.

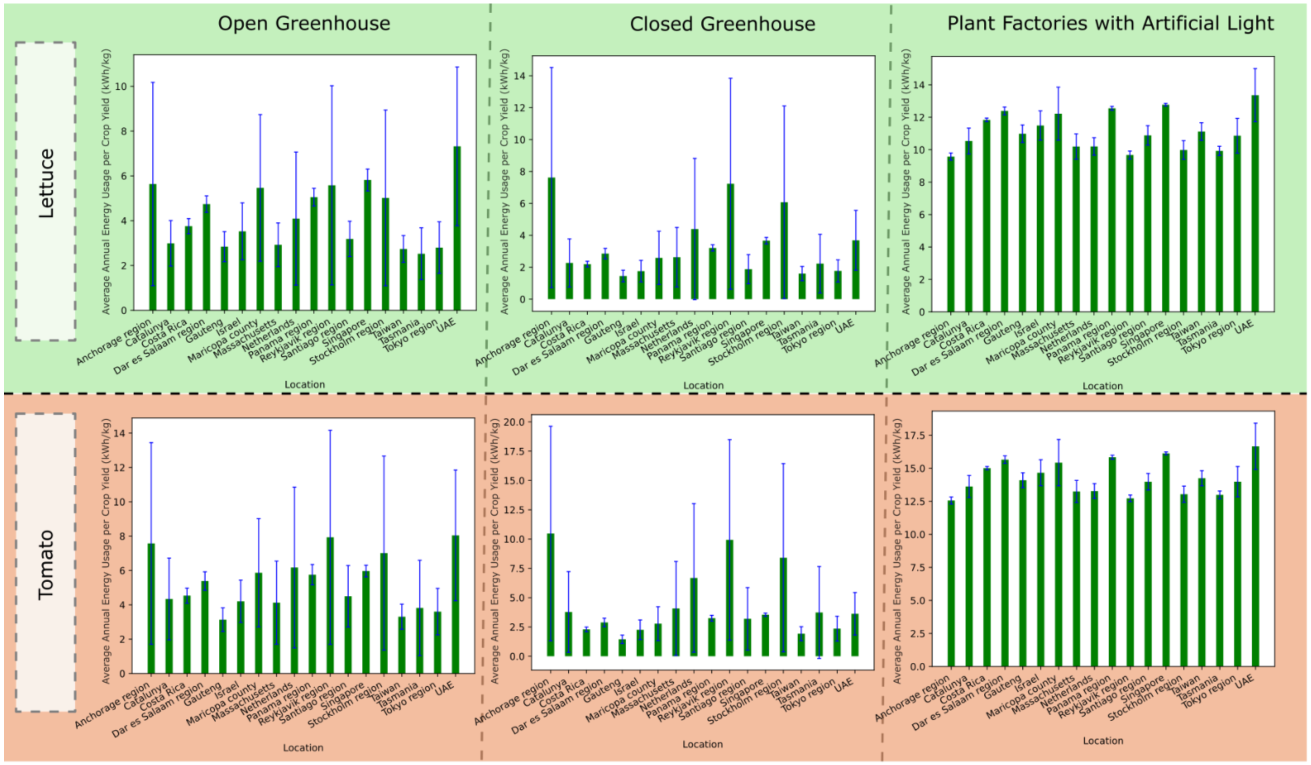

Köppen-Geiger Climate Classification

Location	Country Code	Köppen climate classification	Location	Country Code	Köppen climate classification
Anchorage	USA	Dsc/ Dsd	Panama region	PAN	Am/Af
Catalunya	ESP	Cfa	Reykjavik region	ISL	Cfb / ET
Costa Rica	CRI	Af	Santiago region	CHL	Csb
Dar es Salaam region	TZA	Aw	Singapore	SGP	Af
Gauteng	ZAF	Cwb	Stockholm region	SWE	Cfb
Israel	ISR	Csa/ Bsh	Taiwan	TWN	Cwa/ Cfa
Maricopa county	USA	Bwh	Tasmania	AUS	Cfb
Massachusetts	USA	Dfa	Tokyo region	JPN	Cfa
Netherlands	NLD	Cfb	United Arab Emirates	UAE	BWh

Supplementary Figure 4 Extracted Energy Use-Productivity Metric of Different CEA Configurations in Different Climatic Conditions to Grow Lettuce and Tomato From Literature. The graph presents the mean and standard deviation of the EUP over twelve months (n=12). The variance in simulated energy use-productivity for different CEA configurations is dependent on the type and climatic conditions they operate in[2]. For both open and closed greenhouse configurations, while the mean performance is better than plant factories, the high variability due to seasons implies that for certain countries and certain seasons, plant factories might be a better choice. And because of this variability, certain months or seasons of operation could achieve both food security and sustainability objectives, while cold winter months would be more carbon-emitting than importing it.

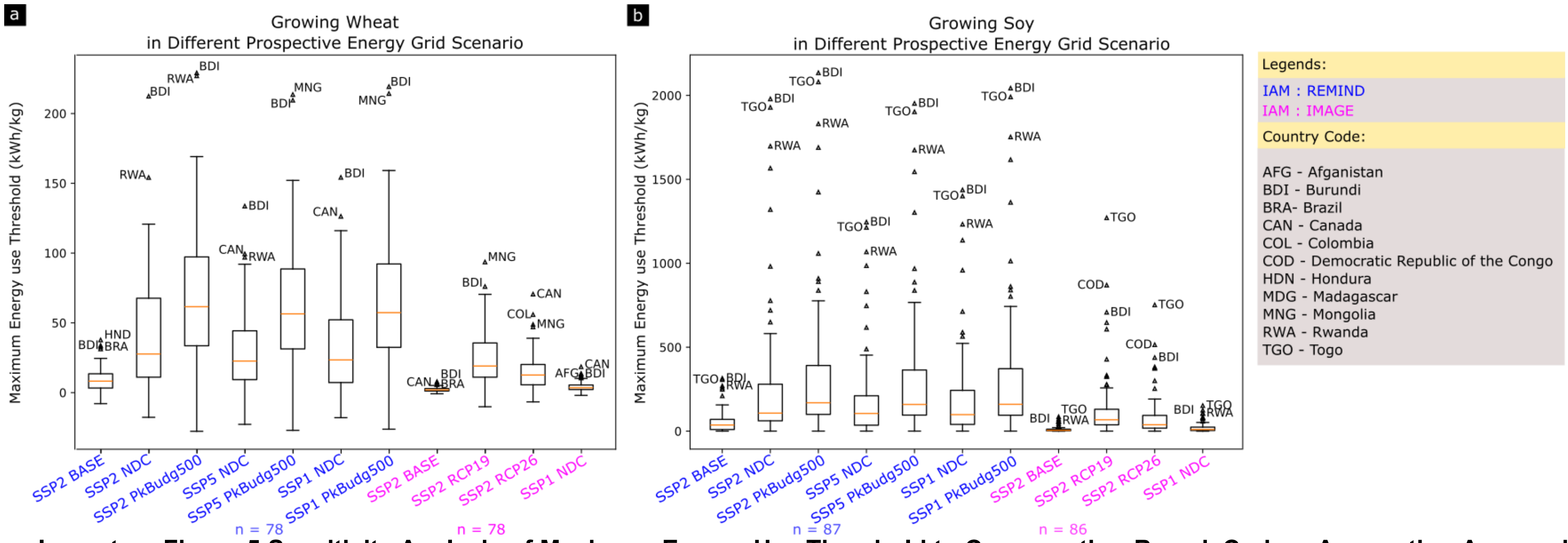

Supplementary Figure 5 Sensitivity Analysis of Maximum Energy Use Threshold to Consumption-Based, Carbon Accounting Approach (Wheat and Soy)

The boxplot reflects the median (blue center bar), interquartile range (± 25% of median), whiskers (1.5 times the interquartile range), and outliers (triangle markers outside of whisker cap). While territorial/ production-based emissions are used by nations to report emissions, consumption needs are met by complex global supply chains. To avoid outsourcing or offshoring of pollutions (carbon leakage), consumption-based emissions help to account for the impact of consumption patterns (import and export) and work towards global decarbonisation. Here, the import of carbon opportunity cost by crop importing country (incurred by agricultural land use in producing country) inherits a heavier carbon emission responsibility in decarbonisation. Hence, for countries with high level of imports from producing countries that incurs high carbon opportunity cost due to high potential of net primary productivity of native vegetation, these countries would pose as favourable locations where CEA would play a bigger role in global decarbonisation.

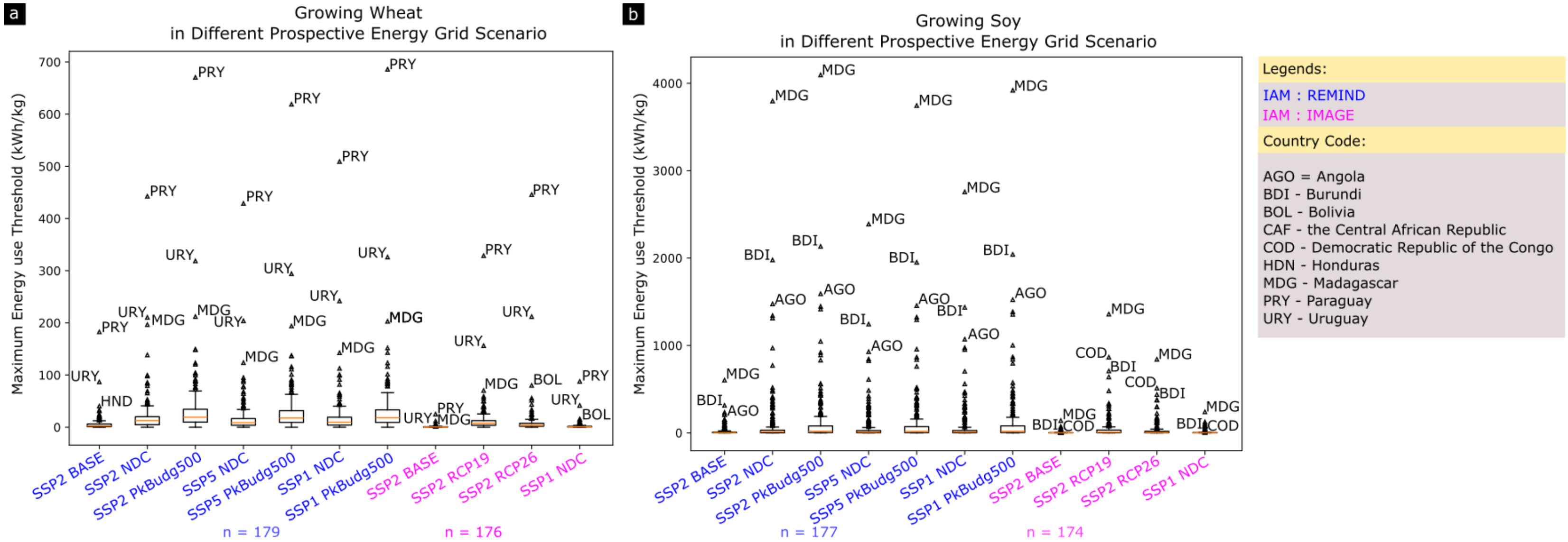

Supplementary Figure 6 Sensitivity Analysis of Maximum Energy Use Threshold to Carbon Opportunity Cost Calculation Approach
The boxplot reflects the median (blue center bar), interquartile range (± 25% of median), whiskers (1.5 times the interquartile range), and outliers (triangle markers outside of whisker cap). While carbon opportunity costs were calculated based on prospective carbon credits from agricultural land use change back to carbon sequestering land use because of shifting agricultural activity onto urban or non-arable land, exclusive use of land saved for climate mitigation purposes might not always be realised or maintained for long periods of time. Hence, an alternative method of accounting carbon opportunity cost would be based on existing land use expansion, where carbon loss from soil and native vegetation was calculated. Here, carbon loss of native vegetation from "newly" expanded agricultural land use between Year 2000 to 2010 was accounted for.

References

1. Wernet, G. *et al.* The ecoinvent database version 3 (part I): overview and methodology. *Int J Life Cycle Assess* **21**, 1218–1230 (2016).
2. Weidner, T., Yang, A. & Hamm, M. W. Energy optimisation of plant factories and greenhouses for different climatic conditions. *Energy Convers Manag* **243**, 114336 (2021).
