# Supplementary material for: Contextual conditions define maximum energy-use threshold in low-carbon controlled environment agriculture for agri-food transformation
Source: Nat Commun. 2026 Feb 2;17:880. doi: 10.1038/s41467-026-68631-w (PMC12865188; doi:10.1038/s41467-026-68631-w)
Supplement: Supplementary file 2 — Description of Additional Supplementary Files [file 41467_2026_68631_MOESM2_ESM.pdf]

Description of Additional Supplementary Files

Supplementary Data 1: Calculated Maximum Criterion for Importing Countries.
