# Supplementary material for: Contextual conditions define maximum energy-use threshold in low-carbon controlled environment agriculture for agri-food transformation
Source: Nat Commun. 2026 Feb 2;17:880. doi: 10.1038/s41467-026-68631-w (PMC12865188; doi:10.1038/s41467-026-68631-w)
Supplement: Supplementary file 4 — Reporting Summary [file 41467_2026_68631_MOESM4_ESM.pdf]

nature portfolio

Corresponding author(s): Shiwei Ng; S Viswanathan

Last updated by author(s): Jan 6, 2026

Reporting Summary

Nature Portfolio wishes to improve the reproducibility of the work that we publish. This form provides structure for consistency and transparency in reporting. For further information on Nature Portfolio policies, see our Editorial Policies and the Editorial Policy Checklist.

Statistics

For all statistical analyses, confirm that the following items are present in the figure legend, table legend, main text, or Methods section.

n/a	Confirmed	
☐	☒	The exact sample size (*n*) for each experimental group/condition, given as a discrete number and unit of measurement
☒	☐	A statement on whether measurements were taken from distinct samples or whether the same sample was measured repeatedly
☒	☐	The statistical test(s) used AND whether they are one- or two-sided<br>*Only common tests should be described solely by name; describe more complex techniques in the Methods section.*
☒	☐	A description of all covariates tested
☒	☐	A description of any assumptions or corrections, such as tests of normality and adjustment for multiple comparisons
☐	☒	A full description of the statistical parameters including central tendency (e.g. means) or other basic estimates (e.g. regression coefficient) AND variation (e.g. standard deviation) or associated estimates of uncertainty (e.g. confidence intervals)
☒	☐	For null hypothesis testing, the test statistic (e.g. *F*, *t*, *r*) with confidence intervals, effect sizes, degrees of freedom and *P* value noted<br>*Give P values as exact values whenever suitable.*
☒	☐	For Bayesian analysis, information on the choice of priors and Markov chain Monte Carlo settings
☒	☐	For hierarchical and complex designs, identification of the appropriate level for tests and full reporting of outcomes
☒	☐	Estimates of effect sizes (e.g. Cohen's *d*, Pearson's *r*), indicating how they were calculated

Our web collection on statistics for biologists contains articles on many of the points above.

Software and code

Policy information about availability of computer code

Data collection	No software was used.
Data analysis	Scripts performing the computation are written in Python programming language and are available at Code Ocean, under the following link: https://doi.org/10.24433/CO.2717777.v1. This includes all the calculation needed to calculate the Maximum Energy use Threshold and generate the results visualised in Figure 3, Figure 5a, Figure 5b, Figure 6a, 6b and swarm plot in 6c. Upon requests, the scripts creating the supplementary figures visualisations will be made available as well. The referenced data are the same output data available in Code Ocean after running the published code. The following software and packages are used: jupyter1.1.1; pandas2.2.3; numpy1.26.; country-converter1.3; countryinfo0.1.2; faostat1.1.2; gdal3.0.4; geopandas1.0.1; geopy2.4.1; kgcpy1.1.8; matplotlib3.9.4; pycountry24.6.1; rasterio1.4.3; rioxarray0.15.0; scipy1.13.1; setuptools57.5.0; shapely2.0.7; xarray-spatial0.4.0

For manuscripts utilizing custom algorithms or software that are central to the research but not yet described in published literature, software must be made available to editors and reviewers. We strongly encourage code deposition in a community repository (e.g. GitHub). See the Nature Portfolio guidelines for submitting code & software for further information.

Data

Policy information about availability of data

All manuscripts must include a data availability statement. This statement should provide the following information, where applicable:

- Accession codes, unique identifiers, or web links for publicly available datasets
- A description of any restrictions on data availability
- For clinical datasets or third party data, please ensure that the statement adheres to our policy

The raw data and generated data in this study have been deposited in the Code Ocean database under the following link: https://doi.org/10.24433/CO.2717777.v1. The estimated energy use data for plant factories and greenhouses under different climatic conditions are available upon request because it is the work published by Weidner, T. at al. Access can also be obtained at the original, cited publication from the original authors. Supplementary Figure 1 to Supplementary Figure 6, and Supplementary Data 1 are available as Supplementary Materials.

Research involving human participants, their data, or biological material

Policy information about studies with human participants or human data. See also policy information about sex, gender (identity/presentation), and sexual orientation and race, ethnicity and racism.

Reporting on sex and gender	n/a
Reporting on race, ethnicity, or other socially relevant groupings	n/a
Population characteristics	n/a
Recruitment	n/a
Ethics oversight	n/a

Note that full information on the approval of the study protocol must also be provided in the manuscript.

Field-specific reporting

Please select the one below that is the best fit for your research. If you are not sure, read the appropriate sections before making your selection.

☐ Life sciences ☐ Behavioural & social sciences ☒ Ecological, evolutionary & environmental sciences

For a reference copy of the document with all sections, see nature.com/documents/nr-reporting-summary-flat.pdf

Ecological, evolutionary & environmental sciences study design

All studies must disclose on these points even when the disclosure is negative.

Study description	The study uses import, export and production data of various crops from FAO to determine the Maximum Energy use Threshold for producing crops of interests using Controlled Environment Agriculture, such that it will be a lower-carbon option to importing or local production.
Research sample	n/a
Sampling strategy	All countries reporting to FAO on their import, export and production data of lettuce, tomato, strawberries, wheat and soya bean are downloaded.
Data collection	There is no primary data collection. Collected data from FAO are used.
Timing and spatial scale	Data between 2012 to 2022 are taken.
Data exclusions	n/a
Reproducibility	The code executing this study has been published on Code Ocean and is available to download as a capsule for reproducibility.
Randomization	n/a
Blinding	n/a

Did the study involve field work? ☐ Yes ☒ No

Reporting for specific materials, systems and methods

We require information from authors about some types of materials, experimental systems and methods used in many studies. Here, indicate whether each material, system or method listed is relevant to your study. If you are not sure if a list item applies to your research, read the appropriate section before selecting a response.

Materials & experimental systems

n/a	Involved in the study
☒	☐ Antibodies
☒	☐ Eukaryotic cell lines
☒	☐ Palaeontology and archaeology
☒	☐ Animals and other organisms
☒	☐ Clinical data
☒	☐ Dual use research of concern
☒	☐ Plants

Methods

n/a	Involved in the study
☒	☐ ChIP-seq
☒	☐ Flow cytometry
☒	☐ MRI-based neuroimaging

Plants

Seed stocks	n/a
Novel plant genotypes	n/a
Authentication	n/a
